# Evaluation of CBSX Proteins as Regulators of the Chloroplast Thioredoxin System

**DOI:** 10.3389/fpls.2021.530376

**Published:** 2021-02-16

**Authors:** Ryota Murai, Yuki Okegawa, Nozomi Sato, Ken Motohashi

**Affiliations:** ^1^Department of Frontier Life Sciences, Faculty of Life Sciences, Kyoto Sangyo University, Kyoto, Japan; ^2^Center for Plant Sciences, Kyoto Sangyo University, Kyoto, Japan

**Keywords:** chloroplast, cystathionine β-synthase X, photo-reduction, photosynthesis, redox regulation, thiol-enzyme, thioredoxin

## Abstract

The chloroplast-localized cystathionine β-synthase X (CBSX) proteins CBSX1 and CBSX2 have been proposed as modulators of thioredoxins (Trxs). In this study, the contribution of CBSX proteins to the redox regulation of thiol enzymes in the chloroplast Trx system was evaluated both *in vitro* and *in vivo*. The *in vitro* biochemical studies evaluated whether CBSX proteins alter the specificities of classical chloroplastic Trx *f* and Trx *m* for their target proteins. However, addition of CBSX proteins did not alter the specificities of Trx *f* and Trx *m* for disulfide bond reduction of the photosynthesis-related major thiol enzymes, FBPase, SBPase, and NADP-MDH. *In vivo* analysis showed that CBSX-deficient mutants grew similarly to wild type plants under continuous normal light conditions and that CBSX deficiency did not affect photo-reduction of photosynthesis-related thiol enzymes by Trx system at several light intensities. Although CBSX proteins have been suggested as modulators in the chloroplast Trx system, our results did not support this model, at least in the cases of FBPase, SBPase, and NADP-MDH in leaves. However, fresh weights of the *cbsx2* mutants were decreased under short day. Since Trxs regulate many proteins participating in various metabolic reactions in the chloroplast, CBSX proteins may function to regulate other chloroplast Trx target proteins, or serve as modulators in non-photosynthetic plastids of flowers. As a next stage, further investigations are required to understand the modulation of Trx-dependent redox regulation by plastidal CBSX proteins.

## Introduction

Proteins belonging to the chloroplastic thioredoxin (Trx) family are reduced *via* ferredoxin and ferredoxin-thioredoxin reductase, using electrons from the photosynthetic electron transport pathway in light conditions (Schurmann and Buchanan, 2008; Geigenberger and Fernie, 2014; Buchanan, 2016). The reduced forms of Trx proteins regulate the activity of thiol enzymes, such as those in the Calvin-Benson cycle, by reduction of their disulfide bonds (Michelet et al., 2013). This Trx-dependent regulation of thiol enzymes is known as chloroplast redox regulation; the NADPH-dependent Trx system in the chloroplast is also known as the NTRC system. NTRC proteins uniquely contain both thioredoxin reductase and thioredoxin domains (Serrato et al., 2004; Perez-Ruiz et al., 2006). NTRC system involvement in plastidial redox-regulation processes has also been suggested (Nikkanen and Rintamaki, 2014; Buchanan, 2016; Cejudo et al., 2019).

Typical Trx family proteins in the chloroplast stroma of *Arabidopsis thaliana* are categorized into five types: two *f*-type isoforms (Trx *f*1 and *f*2); four *m*-type isoforms (Trx *m*1, *m*2, *m*3, and *m*4); one *x*-type isoform (Trx *x*); two *y*-type isoforms (Trx *y*1 and *y*2); and one *z*-type isoform (Trx *z*; Michelet et al., 2013; Serrato et al., 2013; Balsera et al., 2014). Trx *f* and Trx *m* were originally identified as regulators of thiol enzymes involved in photosynthetic carbon fixation and related processes in the chloroplast (Jacquot et al., 1978; Wolosiuk et al., 1979). Furthermore, using *in vitro* biochemical approaches, Trx specificities for their target thiol enzymes were well characterized (Schurmann and Buchanan, 2008; Geigenberger and Fernie, 2014). Calvin-Benson cycle enzymes, including fructose-1,6-bisphosphatase (FBPase; Wolosiuk and Buchanan, 1977; Collin et al., 2003) and sedoheptulose-1,7-bisphosphatase (SBPase; Breazeale et al., 1978; Nishizawa and Buchanan, 1981), and the *γ* subunit of ATP synthase (Schwarz et al., 1997) were mainly regulated by Trx *f*. NADP malate dehydrogenase (MDH) was characterized as an enzyme regulated by Trx *m* (Issakidis et al., 1992), although this enzyme was also later shown to be efficiently activated by Trx *f* (Collin et al., 2003; Yoshida et al., 2015). However, little analysis of the physiological role of the Trx *f* and Trx *m* proteins was available for a long time. Recently, several groups characterized Trx *f*- and Trx *m*-deficient mutants using *Arabidopsis* T-DNA insertion lines and RNA interference. Trx *m*-deficient mutants were impaired in photoreduction of photosynthesis-related enzymes including FBPase, SBPase, and MDH, and showed severe growth defects as a result (Wang et al., 2013; Okegawa and Motohashi, 2015a; Da et al., 2018). In contrast, Trx *f*-deficient mutants did not exhibit substantial defects (Yoshida et al., 2015; Naranjo et al., 2016; Thormahlen et al., 2017), although the *in vitro* biochemical analysis clearly showed that the FBPase (Wolosiuk and Buchanan, 1977) and SBPase (Breazeale et al., 1978) activities were regulated by Trx *f*. These results suggest that *in vivo*, Trx *m* is a more significant redox regulator than Trx *f* in the activation of FBPase, SBPase, and NADP-MDH. Further research is needed to explain the discrepancy between the *in vitro* and *in vivo* studies.

As a regulator of Trx proteins, thioredoxin-interacting protein (TXNIP) was first identified in mammals (Nishiyama et al., 1999). TXNIP interacts with the active site of Trx and inhibits its disulfide reduction activity (Spindel et al., 2012). In plants, CBSX proteins were identified as candidate Trx regulators (Yoo et al., 2011; Jung et al., 2013). CBSX proteins can enhance Trx disulfide reduction activity, in contrast to TXNIP (Yoo et al., 2011). In cyanobacteria, fusion proteins containing a CBS domain and CP12 component were found (Stanley et al., 2013). Plant CP12 protein forms complexes with glyceraldehyde-3-phosphate dehydrogenase (GAPDH) and phosphoribulokinase (PRK), which are also well known redox-regulated Calvin-Benson cycle enzymes. It thus functions as a regulator in the Calvin-Benson cycle (Michelet et al., 2013). The discovery of CBS-CP12 fusion proteins in cyanobacteria implies that the CBS-domain may act as a regulator in the redox regulation system (Lopez-Calcagno et al., 2014). *Arabidopsis* CBSX1 and CBSX2 were indeed proposed as Trx regulators in chloroplasts (Yoo et al., 2011; Jung et al., 2013).

In this study, we evaluated the possibility that CBSX1 and CBSX2 regulate Trx *f* and Trx *m* in the chloroplast redox regulation system using *in vitro* and *in vivo* experiments. Specifically, we investigated whether CBSX1 and CBSX2 modulates the target specificity of Trx *f* and Trx *m* in redox regulation of the photosynthesis-related thiol enzymes FBPase, SBPase, and NADP-MDH. However, we could not find any evidence that support this possibility. CBSX1 and CBSX2 may function not as a Trx regulator for Calvin-Benson cycle enzyme activation, but rather as a regulator for other thiol enzymes in flowers and leaves.

## Materials and Methods

### Construction of Expression System for *Arabidopsis* CBSX1, CBSX2, FBPase, SBPase, and NADP-MDH

The mature forms of *Arabidopsis* chloroplast proteins were predicted using TargetP 1.1 (Emanuelsson et al., 2007). The mature forms of the CBSX1 (AT4G36910), CBSX2 (AT4G34120), FBPase (AT3G54050), SBPase (AT3G55800), and NADP-MDH (AT5G58330) genes were obtained by PCR amplification from an *Arabidopsis* cDNA library (Yamazaki et al., 2004). Primers for cloning and vector constructs are described in Supplementary Table S1. The amplified CBSX1 and CBSX2 genes were directly inserted by seamless cloning into the NdeI and BamHI sites of pET23a and the NcoI and EcoRI sites of pET23d, respectively (vectors from Merck Millipore, Darmstadt, Germany), using seamless ligation cloning extract (SLiCE) from a laboratory *Escherichia coli* JM109 strain (Okegawa and Motohashi, 2015b; Motohashi, 2017b). The amplified FBPase and SBPase genes were cloned into the NdeI and EcoRI sites of pET23a, and the amplified NADP-MDH gene into the NcoI and BamHI sites of pET23d, by the conventional restriction enzyme method. The length of cloned DNA inserts was confirmed by colony PCR (Okegawa et al., 2016; Motohashi, 2017a), using T7 promoter and T7 terminator primers (Okegawa and Motohashi, 2015c). Plasmids were prepared from colony PCR-positive clones and the inserted DNA sequences of plasmids were confirmed by DNA sequencing (Sanger et al., 1977).

### Preparation of *Arabidopsis* CBSX1, CBSX2, FBPase, SBPase, NADP-MDH, Trx *f*1, Trx *m*1, and Trx *m*4 Proteins

*Arabidopsis* proteins were overexpressed in *E. coli* BL21 (DE3; Motohashi et al., 2009).

*Arabidopsis* CBSX1, CBSX2, FBPase, and SBPase proteins were purified as follows. *Escherichia coli* cells overexpressing *Arabidopsis* proteins were suspended in 25 mM Tris-HCl (pH 7.5) and disrupted by sonication (Sonifier® 250, Branson) at 4°C. The disrupted cells were centrifuged at 100,000 *g* for 40 min. The supernatant (crude extract) was applied to a Toyopearl® DEAE-650 M column (Tosoh) and eluted using a linear gradient of NaCl (0–300 mM) in 25 mM Tris-HCl (pH 7.5). Peak fractions containing each *Arabidopsis* protein target were collected, and solid ammonium sulfate was added to obtain a final concentration of 1.4 M. The solution was then loaded into a Toyopearl® Butyl-650 M column (Tosoh) and eluted using an inverse gradient of ammonium sulfate (1.4–0 M) in 25 mM Tris-HCl (pH 7.5). Peak fractions containing each *Arabidopsis* protein were collected. Purified CBSX1 and SBPase were concentrated using Amicon® Ultra-15 (10,000 NMWL membrane, Merck Millipore); the buffer in the protein solution was exchanged with 25 mM Tris-HCl (pH 7.5). Purified CBSX1 and SBPase were stored at −80°C in 16% (v/v) glycerol. The protein fractions of CBSX2 and FBPase underwent further column chromatography for additional purification. CBSX2 collected fractions were dialyzed into 10 mM sodium phosphate buffer (pH 6.8). The solution was then applied to an HA-Ultrogel (Pall) and eluted using a sodium phosphate buffer gradient (pH 6.8, 10–200 mM). His-tagged FBPase collected fractions were dialyzed into 25 mM Tris-HCl (pH 7.5). The solution was then applied to the TALON® metal affinity resin (Takara Bio USA, Inc., Mountain View, CA, United States) equilibrated with 300 mM NaCl and 25 mM Tris-HCl (pH 7.5) and eluted with the buffer, which contained 150 mM imidazole. Purified CBSX2 and FBPase were concentrated and stored at −80°C in 16% (v/v) glycerol.

*Arabidopsis* NADP-MDH was purified without an affinity tag in the absence of dithiothreitol (DTT) as described previously (Okegawa and Motohashi, 2016).

*Arabidopsis* Trx *f*1 (AT3G02730) was purified without an affinity tag as follows (Motohashi and Hisabori, 2006). *Escherichia coli* cells overexpressing *Arabidopsis* Trx *f*1were suspended in 25 mM Tris-HCl (pH 8.1) and disrupted by sonication (Sonifier® 250, Branson) at 4°C. The disrupted cells were centrifuged at 100,000 *g* for 40 min. The supernatant (crude extract) was applied to a Toyopearl® QAE-550C column (Tosoh) and eluted using a linear gradient of NaCl (0–300 mM) in 25 mM Tris-HCl (pH 8.1). Peak fractions containing Trx *f*1 were collected, and solid ammonium sulfate was added to obtain a final concentration of 1.4 M. The solution was then loaded into a Toyopearl® Butyl-650 M column (Tosoh) and eluted using an inverse gradient of ammonium sulfate (1.4–0 M) in 25 mM Tris-HCl (pH 8.1). Peak fractions containing Trx *f*1 were collected and dialyzed into 25 mM MES-NaOH (pH 6.1). The solution was loaded into a Toyopearl® SP-650M column (Tosoh) and eluted using a linear gradient of NaCl (0-300 mM) in 25 mM MES-NaOH (pH6.1). Peak fractions containing Trx*f*1 were collected and then concentrated using Amicon® Ultra-15 (10,000 NMWL membrane); the buffer in the protein solution was exchanged with 25 mM Tris-HCl (pH 7.5). Purified Trx *f*1 was stored at −80°C in 16% (v/v) glycerol.

Trx *m*1 (AT1G03680; Motohashi et al., 2003, 2009) and Trx *m*4 (AT3G15360; Motohashi et al., 2009; Motohashi and Okegawa, 2014) were purified by conventional column chromatography, as described previously, without DTT. Deduced amino acid sequences of Trx *m*1, *m*2, and *m*4 proteins were aligned by ClustalW (Thompson et al., 1994) and formatted by ESPript 3.0 (Robert and Gouet, 2014).

### Preparation of Antibodies

Polyclonal antibodies were prepared from rabbits immunized with CBSX1, CBSX2, FBPase, and SBPase (Okegawa and Motohashi, 2015a), and NADP-MDH (Okegawa and Motohashi, 2016) recombinant proteins. Purified Trx *f*1 and Trx *m*1 were also used as antigens for polyclonal antibodies production. Polyclonal antibodies for PrxA (AT3G11630) and PrxB (AT5G06290) were prepared as peptide antibodies against C-terminal consensus amino acid sequence between PrxA and PrxB (Cys-SMKPDPKLSKEYFSAI). Polyclonal antibodies for PetA (ATCG00540) and PetC (AT4G03280) were prepared from rabbits immunized with partially purified inclusion bodies from recombinant expression proteins (Supplementary Table S1).

### Reduction Assay of Trx-Target Proteins *in vitro*

Thioredoxins-dependent redox-regulated proteins (FBPase, SBPase, and NADP-MDH; 3 μM) were incubated in the presence of DTT (0–1 mM) in 3 mM MgCl_2_ and 25 mM Tris-HCl (pH 7.5) for 30 min at 25°C. Each enzyme was incubated with either Trx-*f* or Trx-*m* alone (total 5 μM), and with one of these plus 3 mM adenosine 5'-monophosphate (AMP) and each of CBSX1 or CBSX2 (total 5 μM). Reactions were terminated by precipitation with trichloroacetic acid (final 10%). The precipitants were washed with ice-cold acetone and dissolved in buffer containing 125 mM Tris-HCl (pH 6.8), 4% sodium dodecyl sulfate (SDS), 20% glycerol, and 2 mM 4-acetamido-4'-maleimidylstilbene-2,2'-disulfonic acid (AMS). The samples were incubated for 30 min at 25°C with vigorous mixing, and then denatured for 2 min at 95°C. Reduced and oxidized proteins were separated by non-reducing SDS-PAGE and stained by Coomassie Brilliant Blue (CBB) R-250.

### Plant Materials and Growth Conditions

*Arabidopsis thaliana* ecotype Columbia-0 was used as the wild type. The T-DNA insertion line GABI_050D12 (*cbsx1*) was obtained from the Nottingham Arabidopsis stock center (NASC, United Kingdom). The T-DNA insertion line SALK_136934C (*cbsx2*; Jung et al., 2013) was obtained from the Salk Institute Genomic Analysis Laboratory (SIGnAL, CA, United States). Plants were grown in soil, or in petri dishes containing Murashige Skoog (MS) medium (Murashige and Skoog, 1962) with 1.2% (w/v) agar and 1% (w/v) sucrose in the growth-chambers (50 μmol photons m^−2^ s^−1^, continuous light, 23°C). Agar-sown seeds were first surface sterilized by soaking in 5% (v/v) sodium hypochlorite for 5 min. To generate double mutants, *cbsx1* and *cbsx2* T-DNA single mutants were crossed. The *cbsx1* and *cbsx2* double mutant was selected from F_2_ plants. The presence of T-DNA insertions was confirmed by PCR (for primer, see Supplementary Table S2) and DNA agarose gel electrophoresis (Motohashi, 2019).

### Analysis of mRNA Expression in *cbsx* Mutants by RT-PCR

The mRNA from wild type and *cbsx* mutants was prepared using Sepasol®-RNA I Super G (Nakalai tesque, Kyoto, Japan), and cDNA samples were synthesized for 20 min at 42°C by ReverTra Ace® (Toyobo, Osaka, Japan), using oligo(dT)_20_. The cDNAs from wild type and *cbsx* mutants were amplified by KAPATaq EXtra (KAPA Biosystems, Wilmington, MA, United States), with the following cycle conditions: 94°C for 2 min, (94°C for 30 s, 55°C for 30 s, and 72°C for 1 min) × 25 cycles. The primers used are listed in Supplementary Table S2. Amplified samples were electrophoresed and visualized by a combination of a black light and a longpass emission-filter (SC-46, Fujifilm, Tokyo, Japan; Motohashi, 2019).

### Expression Analysis of CBSX1, CBSX2, and Redox Regulation-Related Proteins by Western Blotting

To analyze protein expression in *Arabidopsis*, leaves and flowers of wild type and *cbsx* mutants were frozen and ground in liquid nitrogen; total proteins were extracted in 1 × SDS sample buffer [2% (w/v) SDS, 5% (v/v) 2-mercaptoethanol, 10% (v/v) glycerol, and 62.5 mM Tris-HCl (pH 6.8)]. Extracted proteins (30 or 90 μg) were loaded on SDS-PAGE [15% (w/v) acrylamide] and detected by specific antibodies. Immunoblot signal was detected with a LAS-3000UVmini lumino-image analyzer (Fujifilm, Tokyo, Japan). Uncropped original images were indicated in Supplementary Figure S1.

### Chlorophyll Fluorescence Analysis

Chlorophyll fluorescence was measured with a Mini-pulse-amplitude modulation (PAM) portable chlorophyll fluorometer (Walz, Germany). Minimum fluorescence (*F*o) was obtained with open PSII centers in the dark-adapted state by a low-intensity measuring light (wavelength 650 nm, 0.05–0.1 μmol photons m^−2^ s^−1^). A saturating pulse of white light (800 ms, 3,000 μmol photons m^−2^ s^−1^) was applied to determine the maximum fluorescence with closed PSII centers in the dark-adapted state (*F*m) and during actinic light (AL) illumination (*F*m'). The steady-state fluorescence level (*F*s) was recorded during AL illumination (25-828 μmol photons m^−2^ s^−1^). The quantum yield of PSII (*Φ*_PSII_) was calculated as (*F*m'−*F*s)/*F*m' (Genty et al., 1989). The relative rate of electron transport through PSII (ETR) was calculated as Φ_PSII_ × light intensity (μmol photons m^−2^ s^−1^). Non-photochemical quenching (NPQ) was calculated as (*F*m−*F*m')/*F*m'. For the analysis of light-intensity dependence of fluorescence parameters, AL intensity was increased in a stepwise manner every 2 min after the application of a saturating pulse.

### Photo-Reduction Assay of Trx-Target Proteins *in vivo*

Photo-reduction of Trx target enzymes in seedlings was determined using the free thiol-specific modifying reagent, 4-acetamido-4'-maleimidylstilbene-2,2'-disulfonic acid (AMS; Thermo Fisher Scientific, Carlsbad, CA, United States), as described by (Okegawa and Motohashi, 2015a), with minor modifications. Seedlings were incubated in the dark for 3 h and then illuminated at three different light intensities for 1 h (50, 200, or 800 μmol photons m^−2^ s^−1^). Seedlings were collected thereafter and frozen in liquid nitrogen. Frozen samples were ground using 5 mm tungsten beads and incubated in 10 mM AMS, 10 mM EDTA, 4% (w/v) SDS, 8 M urea, and 125 mM Tris-HCl (pH 6.8) for 2 h at 25°C to complete the labeling of thiol groups with AMS. Hydrophilic proteins were separated from the lipid fraction by the addition of an equal volume of chloroform to the sample. The aqueous portion was collected by centrifugation at 15,000 *g* for 10 min). Reduced and oxidized proteins were separated by non-reducing SDS-PAGE [12.5% (w/v) acrylamide] and detected by western blotting. Immunoblot signal was detected with a LAS-3000UVmini lumino-image analyzer (Fujifilm, Tokyo, Japan). The reduction level was quantified using Multi Gauge 3.1 software (Fujifilm, Tokyo, Japan) and the ratio of the reduced form to total protein was determined. Uncropped original images were indicated in Supplementary Figure S1.

## Results

### Contribution of CBSX Proteins to Reduction of Photosynthesis-Related Thiol Enzymes, *in vitro*

**Figure 1:**
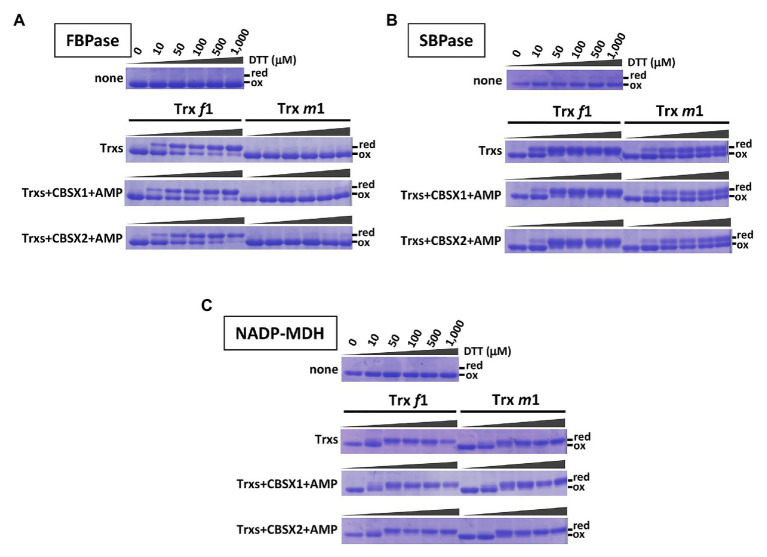
Contribution of cystathionine β-synthase X (CBSX) proteins to thioredoxins (Trx)-dependent reduction of thiol-enzymes, *in vitro*. Thiol-enzyme proteins (3 μM) were reduced in each dithiothreitol (DTT) concentration for 30 min at 25°C. DTT concentrations in each result shown are the same as for the first element. None: contained thiol-enzyme only; Trxs: thiol-enzyme with 5 μM of the indicated Trx; Trxs+CBSXs+AMP: thiol-enzyme with 5 μM indicated Trx, 5 μM CBSX1 or CBSX2 as indicated, and 3 mM adenosine 5'-monophosphate (AMP). Reduced (red) and oxidized (ox) forms of thiol-enzyme were detected by CBB staining. **(A)**: FBPase; **(B)**: SBPase; and **(C)**: NADP-MDH.

Previous work showed that CBSX1 and CBSX2 proteins interact with Trxs (Yoo et al., 2011; Jung et al., 2013), and that CBSX1 protein directly binds with both Trx *f* and Trx *m* proteins *in vitro* (Yoo et al., 2011). Photosynthesis-related thiol enzymes, including several Calvin-Benson cycle enzymes, are regulated by Trx proteins in the light. Published *in vitro* studies indicate preferential reduction by Trx *f* of FBPase (Wolosiuk and Buchanan, 1977; Collin et al., 2003) and SBPase (Breazeale et al., 1978; Nishizawa and Buchanan, 1981), whereas NADP-MDH is reduced by both Trx *f* and Trx *m* proteins (Issakidis et al., 1992; Collin et al., 2003). In contrast, *in vivo*, levels of photo-reduction in thiol enzymes such as FBPase, SBPase, and NADP-MDH were significantly decreased in the Trx *m*-deficient mutants (Okegawa and Motohashi, 2015a). If chloroplast CBSX1 and CBSX2 proteins function as modulators of the chloroplast Trx system, the discrepancy between *in vitro* and *in vivo* results may be explained. To evaluate this possibility, we firstly evaluated the contribution of CBSX proteins to the reduction of thiol enzymes by Trx *f* and Trx *m* proteins, using purified recombinant proteins *in vitro*. Our results were consistent with previous reports described above. FBPase was efficiently reduced *in vitro* by Trx *f*1, not Trx *m*1 (Figure 1A, Trx-only elements), and SBPase was reduced by both Trx *f*1 and Trx *m*1, but Trx *f*1 could reduce more efficiently than Trx *m*1 (Figure 1B, Trx only elements). On the other hand, NADP-MDH was reduced to the same extent by both Trx *f*1 and Trx *m*1 (Figure 1C, Trx only elements). Given this, the effects of addition of CBSX1 or CBSX2 on Trx-dependent reduction of the thiol enzymes by Trx *f*1 and *m*1 were evaluated, *in vitro*. Since CBSX proteins are known to function with AMP as a ligand (Ereno-Orbea et al., 2013), 3 mM of AMP was added to each reaction medium. Under these reaction conditions, both Trx *f*1 and Trx *m*1 were reduced in a DTT concentration-dependent manner (Supplementary Figure S2). Reduction of FBPase and SBPase by Trx *f*1 and Trx *m*1 was not affected by the addition of CBSX1 (Figure 1, CBSX1 elements) or CBSX2 (Figure 1, CBSX2 elements) with AMP. Trx *f*1 reduced FBPase and SBPase disulfide bonds as efficiently in the presence of CBSXs and AMP as with Trx *f*1 alone (Figures 1A,B). The addition of CBSX1 or CBSX2 did not also affect reduction of NADP-MDH by Trx *f*1 and Trx *m*1 with AMP; NADP-MDH was equally reduced by Trx *f*1 and Trx *m*1 in the presence of CBSXs and AMP (Figure 1C). In addition to Trx *m*1, *Arabidopsis* has abundant Trx *m*4 protein in its chloroplasts (Okegawa and Motohashi, 2015a). As with Trx *m*1, reduction patterns of the three thiol enzymes by Trx *m*4 in the presence of CBSX1 or CBSX2 were not affected [Figure 1 (Trx *m*1) and Supplementary Figure S3]. These *in vitro* experiments did not indicate any contribution of CBSX proteins to Trx-dependent reduction of three photosynthesis-related thiol enzymes under studied conditions.

### *Arabidopsis cbsx1* and *cbsx2* Mutants Did Not Show Obvious Visible Defects

**Figure 2:**
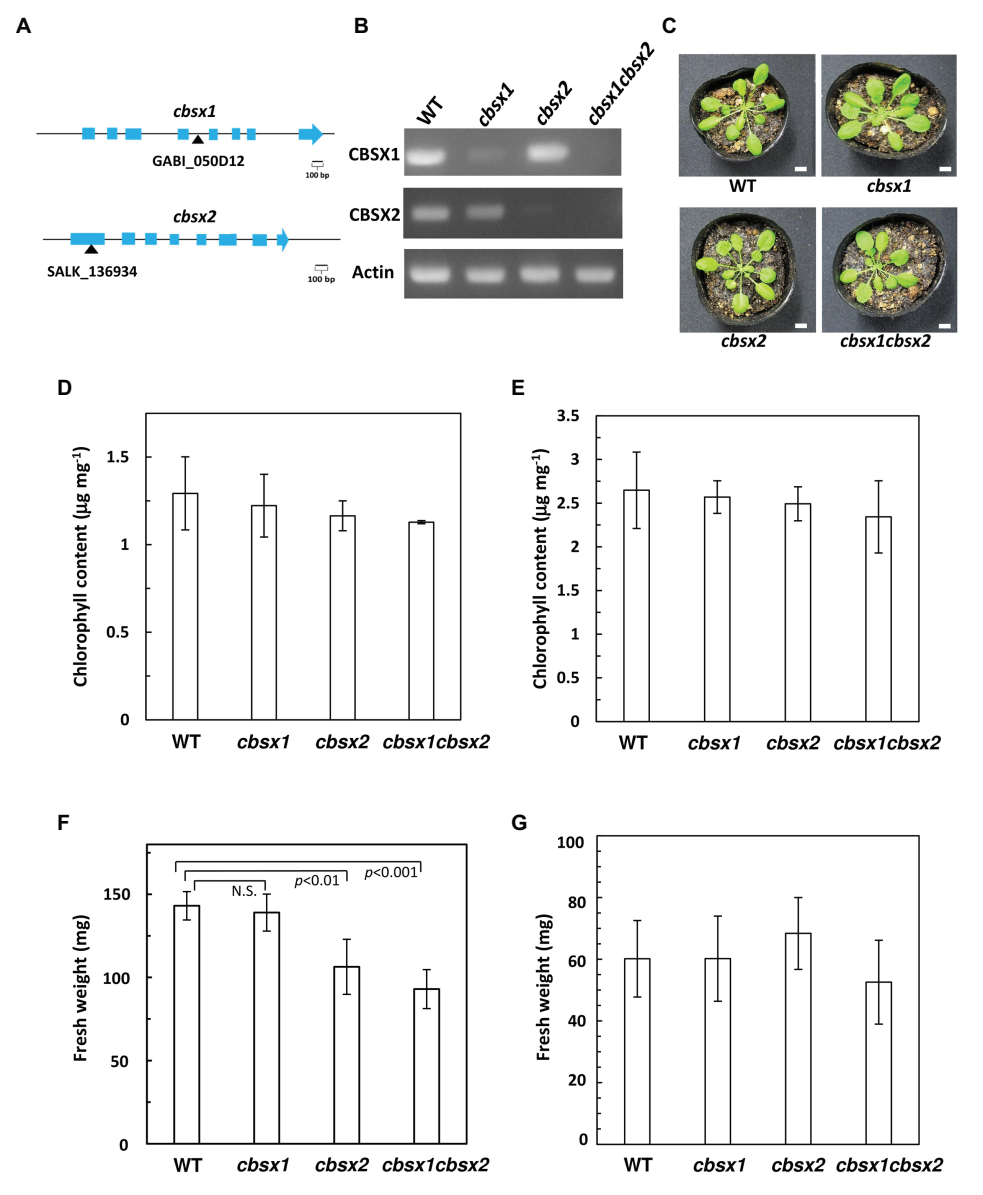
Growth phenotype in *Arabidopsis cbsx* mutants. **(A)** Schematic diagram of CBSX1 and CBSX2 gene structures. The T-DNA insertion sites are indicated with black triangles; light blue boxes indicate coding regions. **(B)** Analysis of mRNA expression in *Arabidopsis cbsx* mutants by RT-PCR. **(C)** Phenotype of *cbsx* mutants grown in soil for 35 days in growth chambers [50 μmol photons m^−2^ s^−1^, short day (8 h light/16 h dark), 23°C]. White bars indicate 10 mm. **(D)** Chlorophyll content of seedlings [50 μmol photons m^−2^ s^−1^, short day (8 h light/16 h dark), 23°C], per unit fresh weight (*n* = 3). **(E)** Chlorophyll content of seedlings (50 μmol photons m^−2^ s^−1^, continuous light, 23°C), per unit fresh weight (*n* = 3). **(F)** Fresh weight of seedlings (*n* = 10) grown on soil for 35 days in growth-chambers [50 μmol photons m^−2^ s^−1^, short day (8 h light/16 h dark), 23°C]. Statistical analyses were performed using the Student’s *t*-test. N.S., not significant. **(G)** Fresh weight of seedlings (*n* = 10) grown on Murashige Skoog (MS) medium for 22 days in growth-chambers (50 μmol photons m^−2^ s^−1^, continuous light, 23°C). Each value is shown as the mean ± SD of independent plants.

*Arabidopsis* has two chloroplast-localized CBSX proteins, namely CBSX1 and CBSX2. The deduced amino acid sequences of these proteins were highly conserved, showing 91% identity. As T-DNA insertion mutants of CBSXs, the *cbsx1* (GABI_050D12), and *cbsx2* (SALK_136934C) mutants were used to investigate the proteins’ physiological significance (Figure 2A). The *cbsx1* and *cbsx2* mutants did not express the mRNAs of *CBSX1* and *CBSX2*, respectively (Figure 2B). To obtain the double mutant *cbsx1 cbsx2*, the *cbsx1* and *cbsx2* single mutants were crossed. The *cbsx* mutants did not show obvious phenotypic defects compared with the wild type under normal growth conditions in soil for 5 weeks (Figure 2C). We could not observe morphological abnormalities (Figure 2C) and differences of chlorophyll content of seedlings (Figures 2D,E) between wild type and the *cbsx* mutants under two different growth conditions. However, the fresh weight of the *cbsx* mutants was gradually decreased in the order of wild type, single mutants, and double mutant in soil under short day condition (Figure 2F). The fresh weight of the *cbsx2* and *cbsx* double mutant grown under short day condition were decreased to ~74% and ~65% of that of wild type (Figure 2F), although there were no obvious differences in the fresh weights between mutants and wild type shoots when these were grown on MS medium agar plates for 3-week-old (Figure 2G).

### *Arabidopsis* CBSX1 and CBSX2 Proteins Are Present in Leaves and Flowers

**Figure 3:**
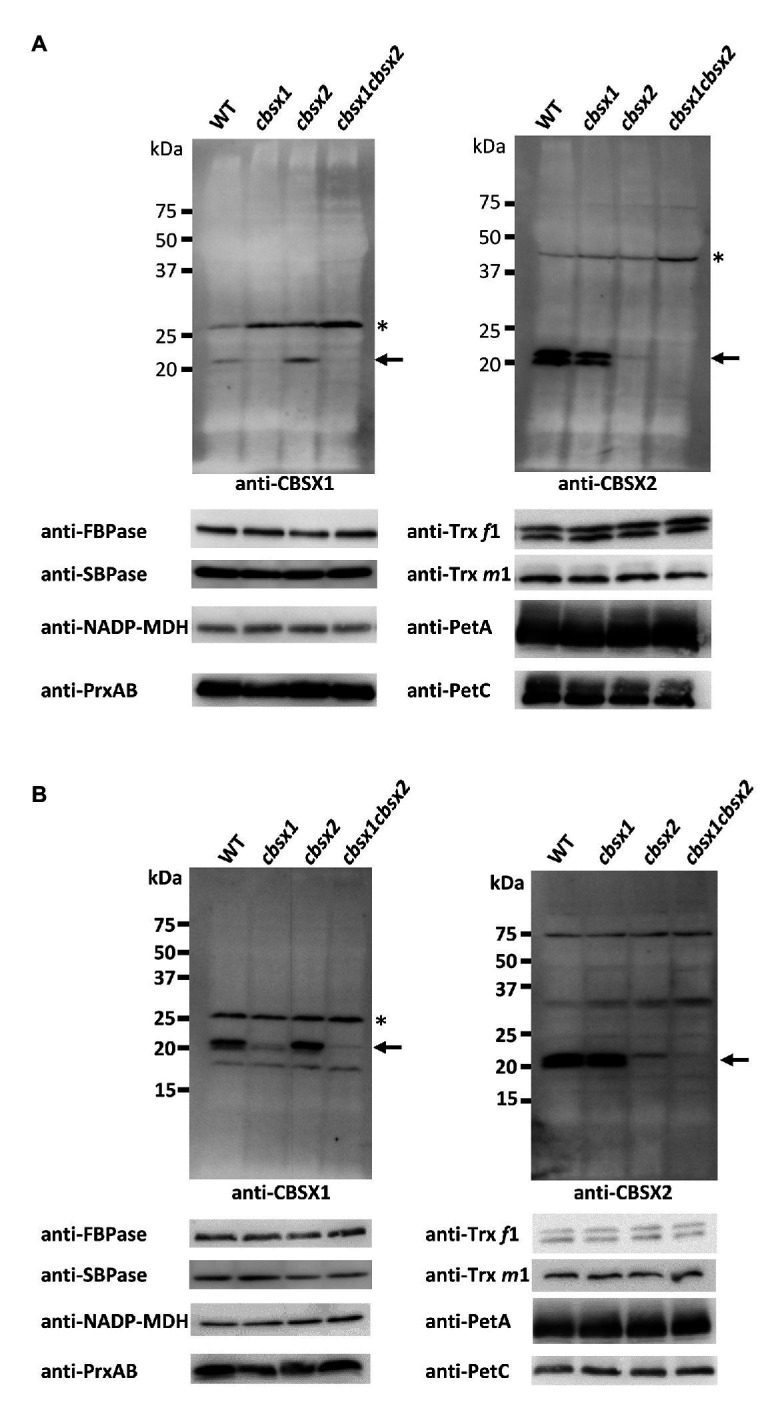
Protein accumulation of CBSX proteins and redox regulation-related proteins in the leaves **(A)** and flowers **(B)** of *cbsx* mutants. Thirty or ninety micrograms of protein were loaded in each lane. Arrows indicate position of CBSX1 or CBSX2; asterisks indicate positions of non-specific bands.

According to the *Arabidopsis* eFP browser 2.0 database for gene expression in *Arabidopsis* (Winter et al., 2007), the mRNA of *CBSX* genes is expressed in leaves, flowers, and seeds. To detect proteins in *Arabidopsis* tissues, we generated specific antibodies for CBSX1 and CBSX2 proteins. The mature forms of the CBSX1 and CBSX2 proteins were predicted to be ~18 kDa proteins, using ChloroP 1.1 (Emanuelsson et al., 1999), and were detected as ~20 kDa proteins in wild type *Arabidopsis*, using antibodies obtained as described above. The specificities of the antibodies for CBSX1 and CBSX2 were confirmed by loss of signal in each deficient mutant (Figure 3). The CBSX1 and CBSX2 proteins were present in leaves (Figure 3A, WT) and flowers (Figure 3B, WT), as suggested by the *Arabidopsis* eFP browser. Western blots detected two bands for CBSX2 protein. We evaluated the two bands as resulting from distinct processing of chloroplast transit sequence elements, not as distinct redox forms, because the CBSX1 and CBSX2 proteins had no cysteine residue in the mature forms. In the *cbsx* deficient mutants, chloroplast Trxs (Trx *f*1 and *m*1), Trx-regulated thiol enzymes (FBPase, SBPase, and NADP-MDH), and PrxA and PrxB that were supplied reducing equivalents by Trxs, were normally detected in leaves (Figure 3A; Supplementary Figure S4). These results indicated that the disappearance of CBSX proteins did not affect the abundance of Trx-related proteins in *Arabidopsis* photosynthetic leaves.

### The *cbsx* Single and Double Mutants Showed Normal Electron Transport Activities in Leaves

**Figure 4:**
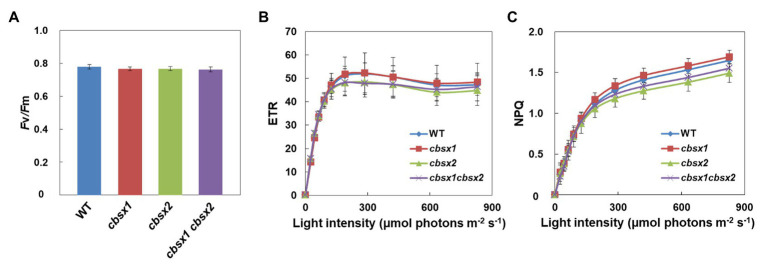
Electron transport activity in the wild type (WT), *cbsx1*, *cbsx2*, and *cbsx1 cbsx2* mutants. **(A)** The maximum quantum yield of PSII (*F*v*/F*m). Each value is the mean ± SD of 10 independent replicates. **(B)** Light-intensity dependence of the electron transport rate (ETR). The ETR was calculated as *Φ*_PSII_ × light intensity (μmol photons m^−2^ s^−1^). Each value is the mean ± SD of five independent replicates. **(C)** Light-intensity dependence of the non-photochemical quenching (NPQ) of chlorophyll fluorescence. Each value is the mean ± SD of five independent replicates. Seedlings were grown in soil for 34 days in growth-chambers (50 μmol photons m^−2^ s^−1^, continuous light, 23°C).

CBSX1 and CBSX2 proteins are suggested to function as modulators of Trxs in leaves, which regulate their target enzyme activity (Yoo et al., 2011; Lopez-Calcagno et al., 2014). Several Calvin cycle enzymes are Trx-regulated proteins. If CBSX proteins are functional regulators for Trxs, photosynthetic activity in leaves may be affected in *cbsx* mutants. To characterize photosynthetic electron transport activity in the mutants, their chlorophyll fluorescence parameters were analyzed (Figure 4). The maximum quantum yield of PSII (*F*v/*F*m) reflects the intactness of PSII. The three *cbsx* mutants exhibited the same level of *F*v/*F*m as the wild type (Figure 4A). The light-intensity dependence of the electron transport rate (ETR) is an indicator of the relative flow rate of electrons through PSII during steady-state photosynthesis, and the NPQ of chlorophyll fluorescence mainly reflects the level of thermal dissipation of excess light energy. No differences in ETR and NPQ were observed between the wild type and the *cbsx* mutants (Figures 4B,C). These results showed that a deficiency of CBSX did not affect these photosynthetic pathways.

### Normal Photo-Reduction of Photosynthesis-Related Thiol Enzymes in *cbsx* Mutants

**Figure 5:**
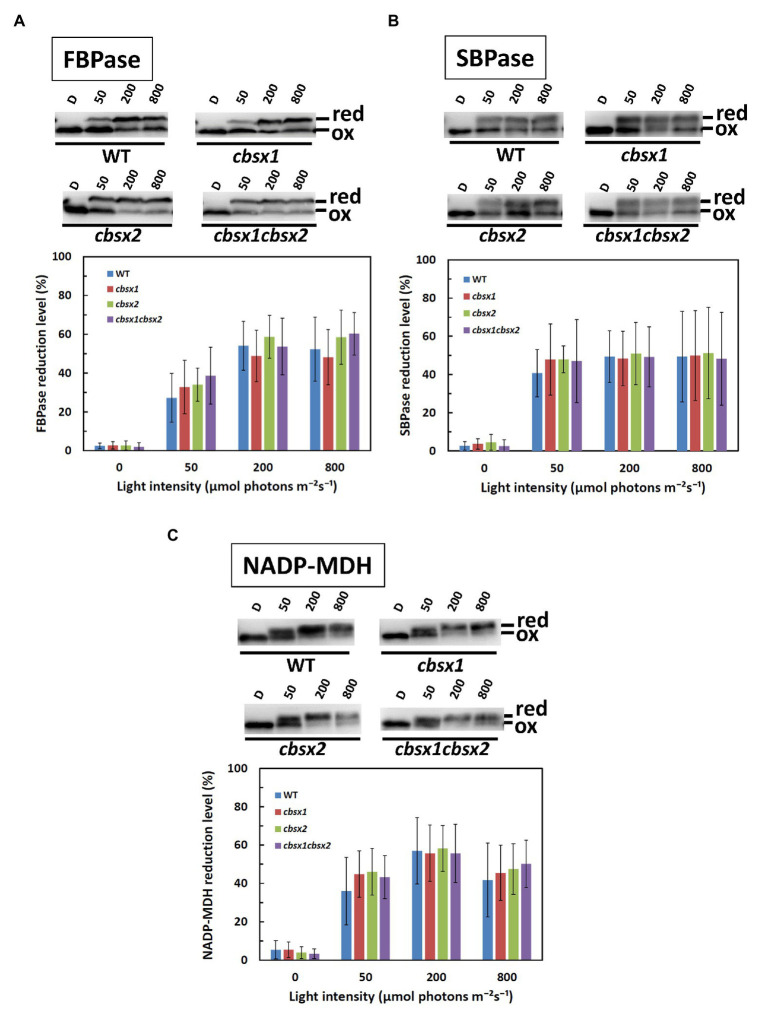
Photo-reduction of photosynthesis-related thiol enzymes in the wild type (WT) and the *cbsx* mutants. **(A)**: FBPase; **(B)**: SBPase; and **(C)**: NADP-MDH. Seedlings were left in darkness for 3 h and illuminated at 50, 200, and 800 μmol photons m^−2^ s^−1^ steps for 1 h each. Samples were collected at each of these light intensities and modified with 4-acetamido-4'-maleimidylstilbene-2,2'-disulfonic acid (AMS). Ninety micrograms of protein samples were loaded on non-reducing SDS-PAGE. Reduced (red) and oxidized (ox) forms were detected by western blotting (upper element of each panel), and reduction levels of thiol enzymes were determined as described in Materials and Methods (lower element of each panel). Each value is the mean ± SD (independent-biological replicates, FBPase: *n* = 9; SBPase: *n* = 8; and NADP-MDH: *n* = 7).

We next investigated the contributions of CBSX1 and CBSX2 to Trx-dependent redox regulation in chloroplasts, using the *cbsx1*, *cbsx2*, and *cbsx1 cbsx2* mutants. Photosynthesis-related thiol enzymes, including several Calvin-Benson cycle enzymes, are reduced by Trx, using electrons from photosynthetic electron transport, and the enzymes are thus activated in light conditions (Hisabori et al., 2007; Michelet et al., 2013; Geigenberger and Fernie, 2014; Buchanan, 2016). If CBSX proteins modulate the activity of photosynthesis-related thiol enzymes *via* a Trx-dependent system, the photo-reduction levels of thiol enzymes should be altered in the *cbsx* mutants. FBPase, SBPase, and NADP-MDH disulfide bonds were reduced by Trx in a light intensity-dependent manner in the wild type (Figure 5, WT). The photo-reduction of FBPase, SBPase, and NADP-MDH in the *cbsx* mutants was compared to that in the wild type (Figure 5, upper elements). At all light intensities studied, the photo-reduction levels of FBPase, SBPase, and NADP-MDH did not differ between the wild type and *cbsx* mutants (Figure 5, lower elements). Additionally, Trxs (Trx *f* and Trx *m*) and Trx-related proteins [FBPase, SBPase, NADP-MDH, 2-Cys peroxiredoxin A (PrxA), and PrxB] were normally present in leaves of the three *cbsx* mutants (Figure 3A). This *in vivo* analysis thus showed no observable contribution of CBSXs to redox regulation of FBPase, SBPase, and NADP-MDH under the studied conditions.

## Discussion

*Arabidopsis* CBSX proteins are proposed as modulators of Trx activity in previous works (Yoo et al., 2011; Jung et al., 2013). Although direct interactions between Trxs and CBSXs have been reported (Yoo et al., 2011), their regulatory mechanism is still unknown. CP12 protein forms the PRK/GAPDH/CP12 regulatory complex in Trx-dependent redox regulation. In cyanobacteria, a fused CP12-CBSX gene was also found (Stanley et al., 2013). These fusion genes imply that CBSX proteins may function as a modulator that cooperates with CP12 in Trx-dependent redox regulation. Lopez-Calcagno et al. (2014) suggested that CBSX proteins are modulators in the redox regulation of Trx-dependent thiol-enzymes in *Arabidopsis* and cyanobacteria (Lopez-Calcagno et al., 2014). However, few biochemical and physiological analyses of CBSX proteins in Trx-dependent redox regulation have been performed. In particular, their contribution to photosynthesis-related Calvin cycle thiol enzyme reduction in leaves has not been tested. In this study, we evaluated the contributions of CBSX1 and CBSX2 proteins to the redox regulation of photosynthesis-related thiol enzymes by chloroplast Trx *f* and Trx *m*, *in vitro* and *in vivo*.

*In vitro*, we evaluated whether CBSX proteins can alter the target specificity of Trx *f* and Trx *m*, using photosynthesis-related thiol enzymes. We could not demonstrate that CBSX1 and CBSX2 proteins modulated the Trx *f*- or Trx *m*-dependent reduction of thiol enzymes such as FBPase, SBPase, and NADP-MDH. CBSX proteins may contribute to the activation of thiol enzymes other than these, because the chloroplast Trx system does function in various redox regulation pathways (Motohashi et al., 2001; Buchanan and Balmer, 2005). Alternatively, other ligands for CBS-domain proteins may be required. CBS-domain proteins are known in wide variety of organisms, and require AMP as a ligand for activation (Baykov et al., 2011). However, several other ligands, including ADP, ATP, and NADH, have been also suggested for CBSXs (Ereno-Orbea et al., 2013). In this study, we used AMP, as the best-characterized ligand. Under these conditions, CBSX proteins had no observable effect on Trx *f* or Trx *m*-dependent activation of photosynthesis-related proteins, but other compounds may be bound as ligands to *Arabidopsis* CBSX1 and CBSX2 proteins in chloroplast redox regulation. *Arabidopsis* has also Trx *m*2 protein, in addition to Trx *m*1 and *m*4, as a major *m*-type Trx. Trx *m*2 is closely related to Trx *m*1 (81% identity in their deduced amino acid sequences, Supplementary Figure S5) and has the same redox potential value (−335 mV) and similar enzyme kinetic parameters (Yoshida and Hisabori, 2017), although Trx *m*4 has lower similarity to Trx *m*1 (60% identity of their deduced amino acid sequences, Supplementary Figure S5) and a different redox potential value (−312 mV). From these facts, we concluded that Trx *m*2 has similar properties to Trx *m*1 proteins and is unlikely to be changed due to specificity by CBSX proteins.

*In vivo*, the *cbsx1*, *cbsx2*, and *cbsx1 cbsx2* mutants did not exhibit obvious morphological defects compared to the wild type (Figure 2C). Consistent with their phenotype, the photosynthetic parameters of the three *cbsx* mutants did not differ from those of the wild type (Figure 4). Deficiencies of the CBSX proteins did not affect photosynthetic activity. Moreover, we analyzed photoreduction of three thiol enzymes, FBPase, SBPase, and NADP-MDH, in three *cbsx* mutants. The photo-reduction rates of these enzymes in the *cbsx* mutants were similar to those of the wild type (Figure 5). For these photosynthesis-related enzymes, the CBSX proteins may not be required for regulation of their activities by Trx *f* or Trx *m* under the light conditions used in this study. However, fresh weight of the *cbsx* single and double mutants was gradually decreased in soil under short day condition (Figure 2F). Particularly, fresh weight of the *cbsx* double mutant was decreased to ~65% of that of WT during the condition (Figure 2F), although obvious difference in growth of the *cbsx* mutants could not be observed on MS plates under continuous light (Figures 2E,G). Interestingly, regarding other redox-related gene deficient mutants, Lepistö et al. reported that the *ntrc* mutants displayed no phenotype when grown under continuous light, but exhibited a strong growth suppression under short day condition (Lepisto et al., 2009). The *ntrc* mutant grown under short day condition had smaller mesophyll cells and contained fewer chloroplasts than wild type leaves. Similar mechanism may also function in the *cbsx* mutants.

From the *in vitro* and *in vivo* analyses, we concluded that CBSX1 and CBSX2 proteins did not function as modulators of Trx *f* and Trx *m* in redox regulation of the three major thiol enzymes FBPase, SBPase, and NADP-MDH in leaves, at least under the studied conditions. In the last 20 years, a large number of Trx target proteins have been newly identified (Geigenberger et al., 2017). More recently, new target proteins have also been identified (Okegawa and Motohashi, 2020; Yoshida et al., 2020). Although *Arabidopsis* CBSX proteins are suggested to modulate Trx-activity (Yoo et al., 2011; Jung et al., 2013), it is unclear which Trx-dependent pathways do CBSX proteins regulate. There are several possibilities other than the possibility of Trx-related pathways, including Calvin cycle enzymes in the leaves. CBSX proteins may modulate other unknown redox-related pathways, particularly concern to the growth delay of the *cbsx* mutants grown in soil under short day condition. Expression of *CBSX* mRNA was seen in leaves, flowers, and seeds in the *Arabidopsis* eFP Browser 2.0 (Winter et al., 2007) and we detected accumulation of CBSX proteins in leaves and flowers by western blotting, using anti-CBSX1 and anti-CBSX2. Given this, CBSX proteins may function as modulators for thiol enzymes other than those involved in photosynthesis in leaves and flowers. In particular, redox regulation by Trx proteins in flowers remains largely uninvestigated. To elucidate the functions of CBSX proteins, the effects of Trx proteins including Trx *f*, Trx *m*, and other chloroplast Trxs on thiol enzymes in leaves and flowers should form the next stage of investigation.

## Data Availability Statement

The datasets generated for this study are available on request to the corresponding author.

## Author Contributions

YO and KM contributed conception and design of the study. RM, YO, NS, and KM carried out experiments. RM, YO, NS, and KM performed the statistical analysis. RM and KM wrote the first draft of the manuscript. YO and KM wrote sections of the manuscript. All authors contributed to the article and approved the submitted version.

### Conflict of Interest

The authors declare that the research was conducted in the absence of any commercial or financial relationships that could be construed as a potential conflict of interest.
