# Supplementary material for: Evaluation of CBSX Proteins as Regulators of the Chloroplast Thioredoxin System
Source: Front Plant Sci. 2021 Feb 16;12:530376. doi: 10.3389/fpls.2021.530376 (PMC7921703; doi:10.3389/fpls.2021.530376)
Supplement: Supplementary file 1 [file Table_1.pdf]

Table S1 Oligonucleotides for cloning and vector construction

Primer name	Sequence (5′ to 3′)
For CBSX1 (AT4G36910)	
AtCBSX1_mat_F	5'-ttaagaaggagatatacatATGAGTGGAGTGTACACTGTTGG-3'
AtCBSX1_R	5'-gacggagctcgaattcggatccTCAAGCATTCCTATCACCAG-3'
For CBSX2 (AT4G34120)	
AtCBSX2_mat_F-Nco	5'-ttaagaaggagatataccATGGCAAAAAATGGAGGTTACA-3'
AtCBSX2_R	5'-gtcgacggagctcgaattCTATGTAGAGTTCTCGGTTTC-3'
For FBPase (AT3G54050)	
AtFBPaseF	5'-aactgcagcatATGGCCGTAGCGGCGGATGCT-3'
AtFBPaseR-CHis	5'-cggaatTCAATGGTGATGGTGATGGTGATGGTGAGCCAAGTACTTCTCCAGCTT-3'
For SBPase (AT3G55800)	
AtSBPase_F	5'-aactgcagcatATGGCCACAAAAGCTAAGAGCAA-3'
AtSBPase_R	5'-cggaattCTAAGCGGTAACTCCAATGGG-3'
For NADP-MDH (AT5G58330)	
AtcpNADP-MDH_F_Nc	5'-aactgcagaccATGGTTTCTCAAAATAGCCAAGCT-3'
AtcpNADP-MDH_R	5'-cgcggatccTCAAACTTCCCCAGGAAGCAT-3'
For PetA (ATCG00540)	
AtPetAF	5'-aactgcagcatATGTATCCGATTTTTGCCCAGCAG-3'
AtPetAR-His	5'-cggaattCTAATGATGATGATGATGATGATGATGAAAATTCATTTCGGATAATTG-3'
For PetC (AT4G03280)	
AtPetCF	5'-aactgcagcatATGGCGTCGAGTATTCCAGCAGAC-3'
AtPetCR-His	5'-cggaattCTAATGATGATGATGATGATGATGATGAGACCACCATGGAGCATCACC-3'

Open reading frame regions were indicated by capital letters. Introduced restriction enzyme cleavage sites were underlined (NdeI, NcoI, BamHI and EcoRI).

Table S2 Oligonucleotides for T-DNA insertion mutants screening and RT-PCR

Primer name	Sequence (5′ to 3′)
For *cbsx1* (GABI_050D12) screening	
GABI_050D12 LP	5'-CCATTGGTTTTGCTGAGTAGC-3'
GABI_050D12 RP	5'-TTGACGAAGACTGGAAATTGG-3'
For *cbsx2* (SALK_136934C) screening	
SALK_136934C LP	5'-TTTCCAGTACATTGCGTCATG-3'
SALK_136934C RP	5'-CAGAAGTTCCAACGCTGAAAG-3'
For T-DNA insertion screening	
GABI_RB_o3144 (GABI_050D12, *cbsx1*)	5'-GTGGATTGATGTGATATCTCC-3'
LBb1.3 (SALK_136934C, *cbsx2*)	5'-ATTTTGCCGATTTCGGAAC-3'
For CBSX1 RT-PCR	
CBSX1_RT_F1	5'-CGTCCTTTACTCTGTTCCACTC-3'
CBSX1_RT_R1	5'-AGCATCTTCCAGGTTGGTTT-3'
For CBSX2 RT-PCR	
CBSX2_RT_F1	5'-CCCATAACTCGACTTCCACTAC-3'
CBSX2_RT_R1	5'-GCATCAACAACGGGTAATCTTC-3'
For Actin RT-PCR	
Actin_F	5'-GAGAGATTCAGGTGCCCAG-3'
Actin_R	5'-AGAGCGAGAGCGGGTTTTCA-3'
