# Supplementary material for: Evaluation of CBSX Proteins as Regulators of the Chloroplast Thioredoxin System
Source: Front Plant Sci. 2021 Feb 16;12:530376. doi: 10.3389/fpls.2021.530376 (PMC7921703; doi:10.3389/fpls.2021.530376)
Supplement: Supplementary file 2 [file Data_Sheet_1.pdf]

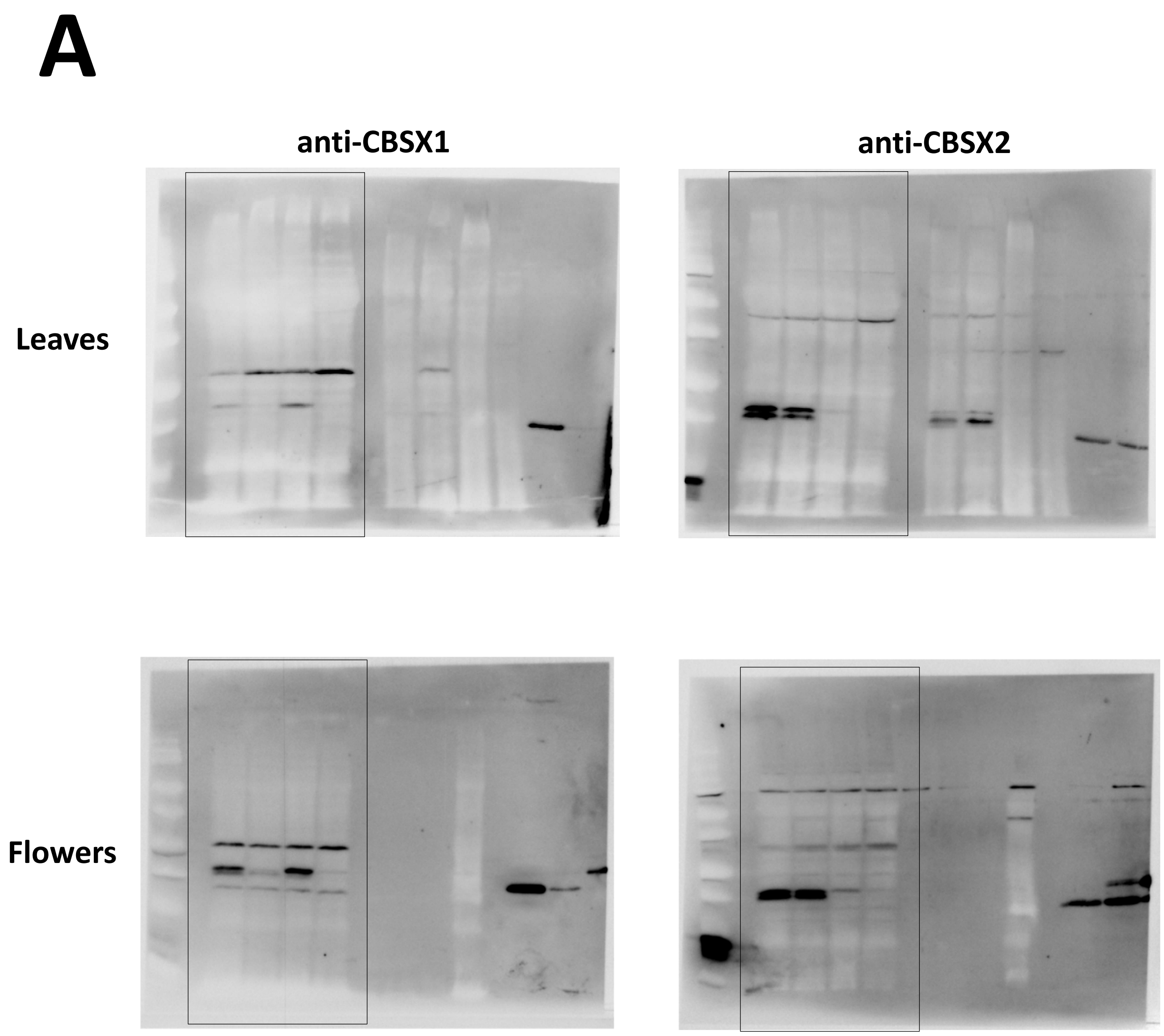

Figure S1A Uncropped images for Figure 3A and 3B (for anti-CBSX1, and -CBSX2).

B

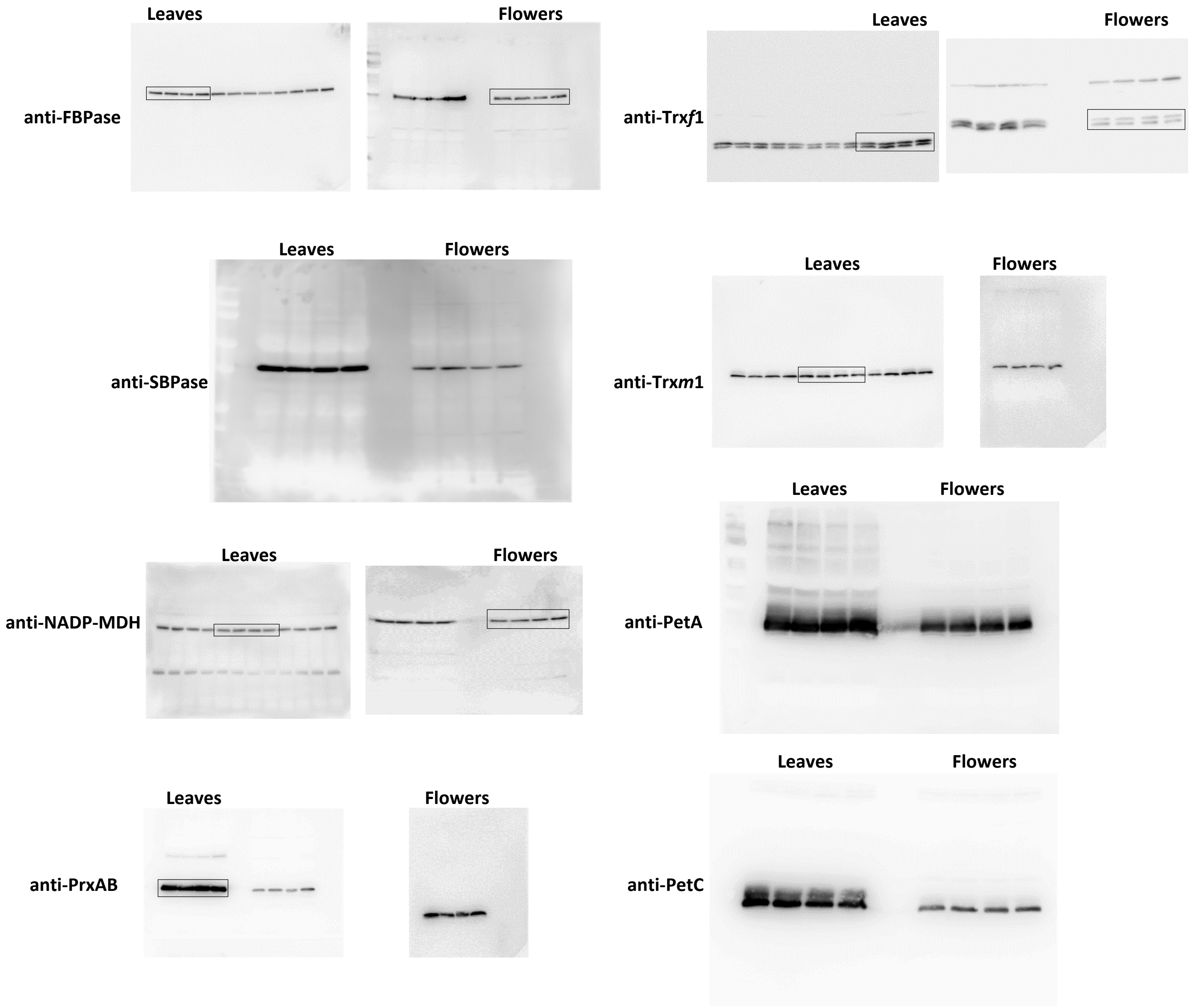

Figure S1B Uncropped images for Figure 3A and 3B (for anti-FBPase, -SBPase, -NADP-MDH, -PrxAB, -Trx*f*1, -Trx*m*1, -PetA, and -PetC).

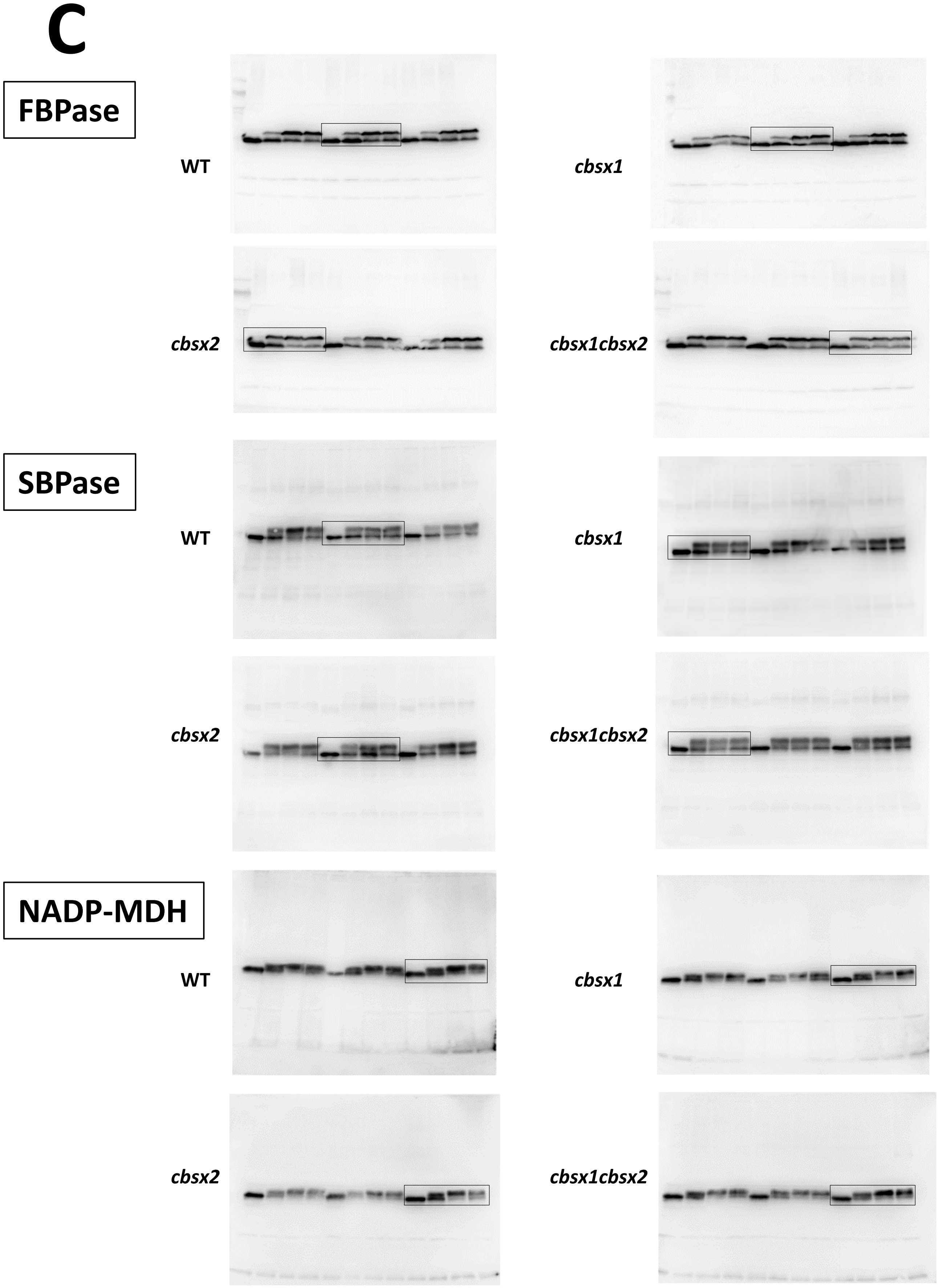

Figure S1C Uncropped images for Figure 5.

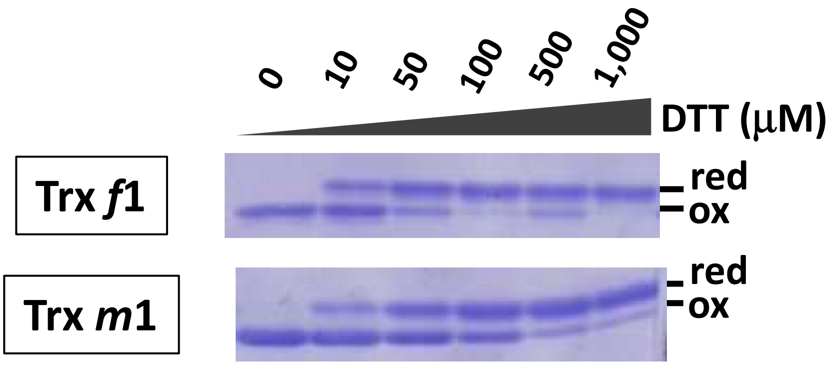

Figure S2 DTT-dependent reduction of Trx *f*1 and Trx *m*1 in the presence of FBPase, CBSX2, and AMP, *in vitro*.

Trxs (5 μM) were reduced in each DTT concentrations for 30 min at 25° C, in the presence of FBPase (3 μM), CBSX2 (5 μM), and AMP (3 mM). Reduced (red) and oxidized (ox) forms of Trx *f*1 or Trx *m*1 were detected by CBB staining.

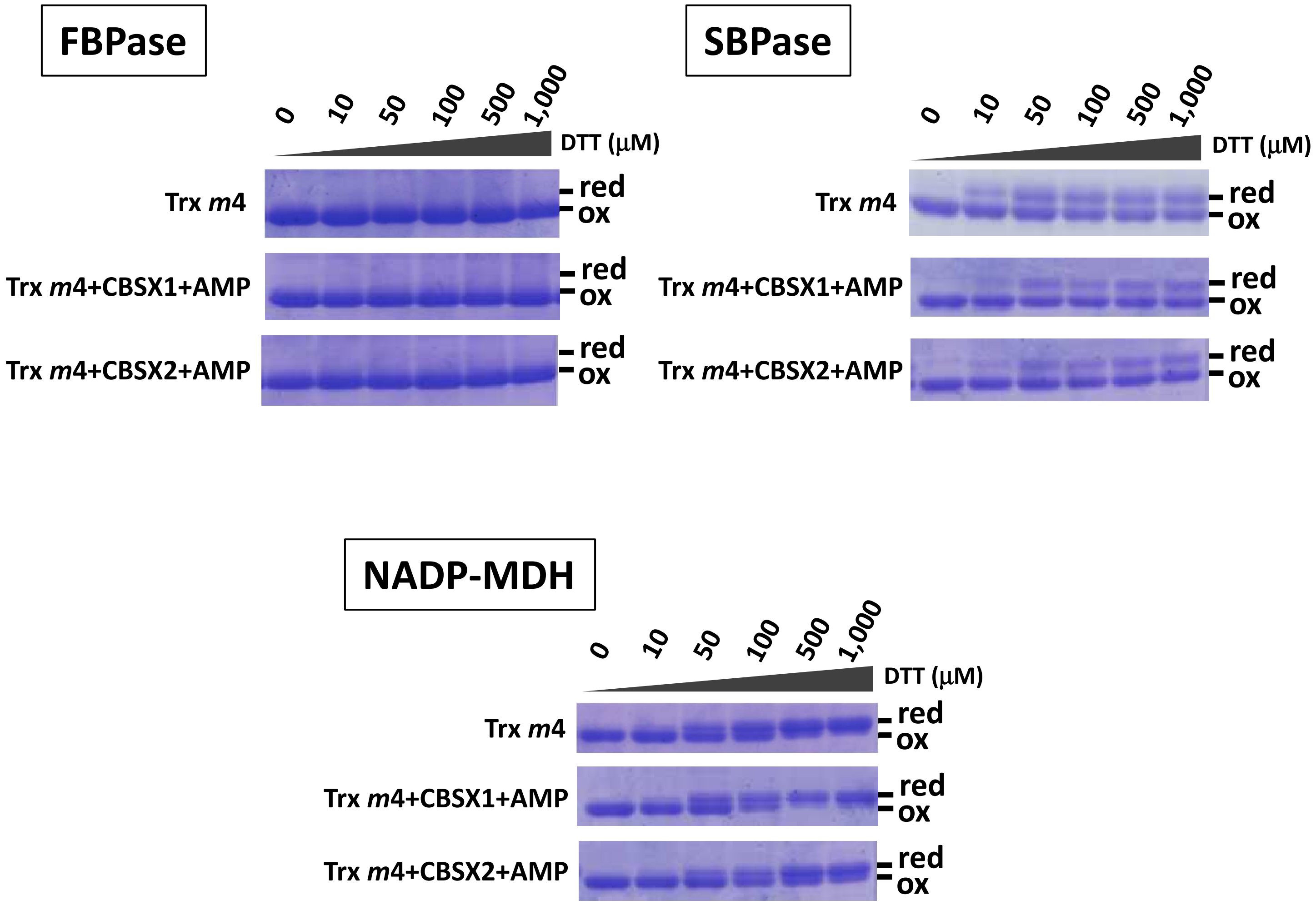

Figure S3 Contribution of CBSX proteins to Trx *m*4-dependent reduction of photosynthesis-related thiol enzymes, *in vitro*.

Thiol enzymes (3 μM) were reduced by Trx *m*4 (5 μM) in each DTT concentration for 30 min at 25° C. Trx *m*4: contained each thiol enzyme with 5 μM Trx *m*4; Trx *m*4+CBSXs+AMP: each thiol enzyme with 5 μM Trx *m*4, 5 μM CBSX1 or CBSX2 as indicated, and 3 mM AMP. Reduced (red) and oxidized (ox) forms of three thiol enzymes were detected by CBB staining.

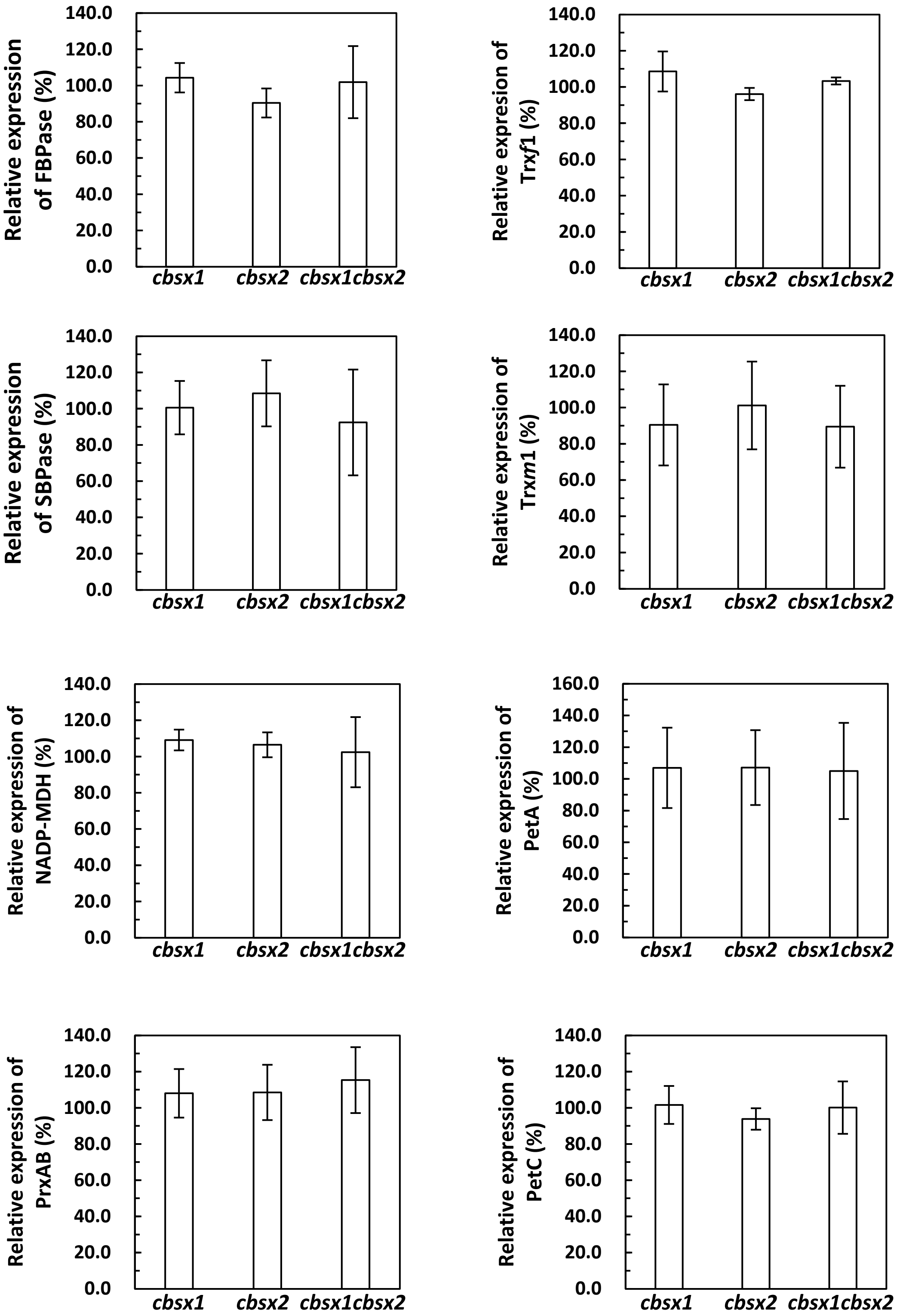

Figure S4 Relative expression levels of redox-related proteins in leaves of the *cbsx* mutants.

Relative expression levels of redox-related proteins in leaves of the *cbsx* mutants were estimated by western blotting using specific antibodies (*n*=3). Expression levels of wild-type was set to 100 (%).

A

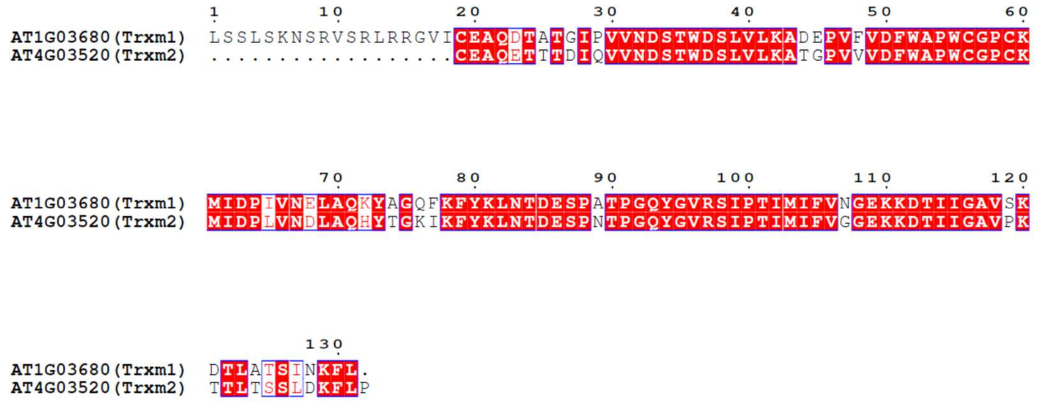

B

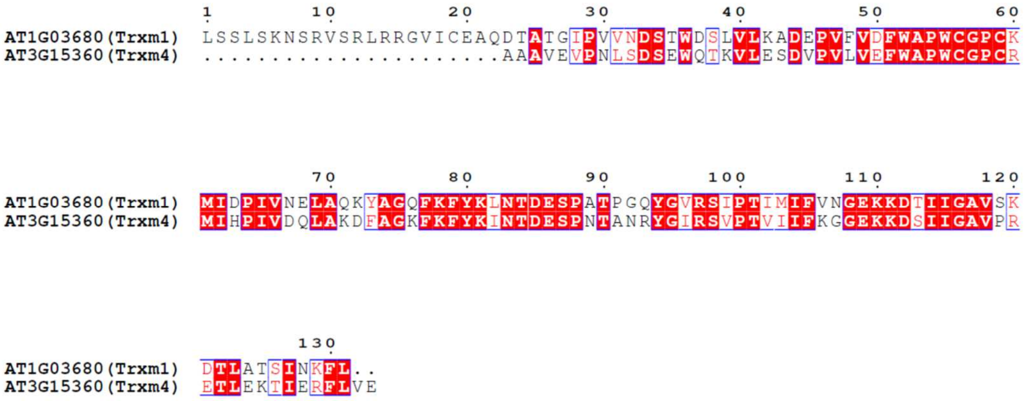

Figure S5 Alignment of deduced amino acid sequences of *Arabidopsis* chloroplast Trx *m*1, *m*2, and *m*4 proteins (mature form).

Arabidopsis Trx *m*1 vs *m*2 (A) and Trx *m*1 vs *m*4 (B) were aligned by ClustalW, and formatted by ESPript 3.0. Identical amino acids (white letters in red) and similar amino acids (red letters in white) were indicated, respectively.
